# Thoracic epidural analgesia reduces gastric microcirculation in the pig

**DOI:** 10.1186/s12871-016-0256-4

**Published:** 2016-10-06

**Authors:** Rikard Ambrus, Rune B. Strandby, Niels H. Secher, Kim Rünitz, Morten B. S. Svendsen, Lonnie G. Petersen, Michael P. Achiam, Lars B. Svendsen

**Affiliations:** 1 Department of Surgical Gastroenterology C, Rigshospitalet, University of Copenhagen, Blegdamsvej 9, DK - 2100 Copenhagen Ø, Denmark; 2 Department of Anesthesiology 2041, Rigshospitalet, University of Copenhagen, Blegdamsvej 9, DK - 2100 Copenhagen Ø, Denmark; 3 Marine Biological Section, University of Copenhagen, Strandpromenaden 5, DK - 3000 Elsinore, Denmark

**Keywords:** Splanchnic microcirculation, Thoracic epidural analgesia, Laser speckle contrast imaging, Pigs

## Abstract

**Background:**

Thoracic epidural analgesia (TEA) is used for pain relief during and after abdominal surgery, but the effect of TEA on the splanchnic microcirculation remains debated. We evaluated whether TEA affects splanchnic microcirculation in the pig.

**Methods:**

Splanchnic microcirculation was assessed in nine pigs prior to and 15 and 30 min after induction of TEA. Regional blood flow was assessed by neutron activated microspheres and changes in microcirculation by laser speckle contrast imaging (LSCI).

**Results:**

As assessed by LSCI 15 min following TEA, gastric arteriolar flow decreased by 22 % at the antrum (*p* = 0.020) and by 19 % at the corpus (*p* = 0.029) of the stomach. In parallel, the microcirculation decreased by 19 % at the antrum (*p* = 0.015) and by 20 % at the corpus (*p* = 0.028). Reduced arteriolar flow and microcirculation at the antrum was confirmed by a reduction in microsphere assessed regional blood flow 30 min following induction of TEA (*p* = 0.048). These manifestations took place along with a drop in systolic blood pressure (*p* = 0.030), but with no significant change in mean arterial pressure, cardiac output, or heart rate.

**Conclusion:**

The results indicate that TEA may have an adverse effect on gastric arteriolar blood flow and microcirculation. LSCI is a non-touch technique and displays changes in blood flow in real-time and may be important for further evaluation of the concern regarding the effect of thoracic epidural anesthesia on gastric microcirculation in humans.

**Trial registrations:**

Not applicable, non-human study.

## Background

Thoracic epidural analgesia (TEA) is used for abdominal surgery to reduce intra- and postoperative pain, improve postoperative respiratory function, and to attenuate the surgical stress response [1]. Also, epidural analgesia has been shown to be associated with reduced risk of postoperative mortality and has beneficial effects on postoperative cardiovascular complications [2]. However, the effect of TEA on the splanchnic microcirculation remains unclear [3] and a concern is whether TEA affects splanchnic blood flow, since poor surgical outcome is correlated to ischemia at the anastomosis [4].

TEA-mediated reduced blood pressure could be of importance for the splanchnic microcirculation, since probes at the mesenteric arteries have demonstrated a TEA-mediated reduced blood flow [5, 6]. However, a reduced splanchnic blood flow in response to TEA has not been confirmed in experimental studies with focus on splanchnic microcirculation and oxygenation [7, 8]. Furthermore, laser Doppler flowmetry indicates increased postoperative gastric mucosal microcirculation with the use of TEA [9]. Thus, the effects of TEA on splanchnic microcirculation are inconsistent and conflicting results likely reflect different approaches to evaluate the microcirculatory flow and oxygenation. For example, both evaluation of tissue oxygenation and laser Doppler flowmetry require contact to the surface of the tissue [7, 9] and that could affect the measurements.

The aim of the present study was to evaluate the effect of TEA on splanchnic microcirculation in pigs with the use of microspheres [10, 11] and laser speckle contrast imaging (LSCI) for non-contact assessment [12]. We hypothesized that TEA would maintain splanchnic microcirculation.

## Methods

The study was conducted under supervision of the veterinarians at the Department of Experimental Medicine, The Panum Institute, University of Copenhagen and was approved by the Danish Animal Experiments Inspectorate (2012-15-2934-00186). According to the replacement, reduction, and refinement principle [13], the current trial was performed after evaluation of the inter-observer reproducibility of LSCI determined microcirculation [12], and followed by another study comparing the microcirculation assessed by the LSCI, microspheres, and indocyanine green technique (Nerup et al., unpublished).

### Anesthesia and monitoring

Nine healthy female pigs (Danish Landrace/Yorkshire; weight 32.0 ± 1.8 kg (mean ± SD, aged 9–10 weeks) were sedated with a standardized mix of intramuscular Zoletil vet. (5 mg/kg), xylazine, ketamine, methadone, and butorphanol. Following placement of venous access in both ears, anesthesia was maintained with propofol (15 mg/kg/h) and fentanyl (5 μg/kg/h). After endotracheal intubation, positive pressure ventilation was established with 25 – 40 % oxygen. Catheters (8 F, Unomedical, Denmark) were then established in the left and right femoral arteries for blood sampling and arterial pressure monitoring. Ventilation was adjusted to an arterial carbon dioxide (PaCO_2_) of 4.5 – 6.5 and an oxygen tension (PaO_2_) of 11–15 kPa, as controlled every 30^th^ min (ABL™ 700; Radiometer Medical ApS, Copenhagen, Denmark). A gastric tube was inserted to empty gastric content and lactated Ringer’s solution was administrated intravenously at 5 ml/kg/h.

An epidural catheter (Epidural Minipack System 1, Smiths Medical, Hranice, Czech Republic) was placed at 7th – 8th or 8th – 9th thoracic intervertebral space using the “loss of resistance” technique. Thereafter, a pulmonary artery catheter (7 F, Edwards, Life Sciences, Irvine, CA, USA) was inserted via the right external jugular vein for determination of central pressures and cardiac output by thermodilution (Vigilance® Monitor, Edwards Critical Care Division, Irvine). Also, a catheter (8 F, Unomedical, Denmark) was placed in the left ventricle of the heart via the left carotid artery. The catheters were flushed with heparinized lactated Ringer’s solution (100 IE/ml) after use. A 10 F catheter (Unomedical, Denmark) was surgically placed in the bladder for monitoring urine output. Hemodynamic data were recorded using Powerlab 16/35 (AD Instruments, Dunedin, New Zealand). All the vascular catheters were placed by dissection, assisted by the veterinarian. The gauzes used during the dissection were weighted and hemoglobin and hematocrit values were taken prior to and after the instrumentation to assess any bleeding.

### Surgical procedure

After instrumentation, the intestines, stomach, and liver were exposed by a midline laparotomy. No pressure or strangulation of the organs was introduced or observed. The liver (segment 3), the small intestine (10 cm from the caecum), and the antrum (3 cm from the pylorus) and the corpus (3 cm from the greater curvature) of the stomach were marked to ensure regions of interest (ROI).

### Experimental protocol

After 15 min of stabilization following the laparotomy, TEA was induced by 2 ml lidocaine/adrenaline (2 %, SAD, Amgros I/S, Denmark), followed by bolus injection of 2 ml bupivacaine (5 mg/ml, SAD) and infusion of 2 ml/h bupivacaine/morphine (2.5 mg/50 μg/ml), i.e., half of the dose used for adult patients taking the weight of the animals into account.

The effect of TEA on the splanchnic microcirculation was assessed three times: at baseline, 15 min, and 30 min following induction of TEA. LSCI was used to determine changes in microcirculation at each ROI. Simultaneously, a suspension of microspheres was injected into the left ventricle of the heart, and blood from the femoral arterial was obtained by automated aspiration (6 ml/min) (Harvard Apparatus PHD 2000 Infusion/Withdraw Pump, MA, USA) starting 30 s prior to the injection and continued for 120 s. This rate was used as reference sample flow when regional blood flow was calculated.

At the end of each trial, the pig was euthanized by intravenous pentobarbital and tissue samples (approximately 1 g) were collected from each ROI. The samples were weighed (Quintix ®, Sartorius, Bie & Berntsen A/S, Denmark) and dried overnight at 70 °C together with the blood samples. After drying, the samples were quantified for microspheres (BioPAL Inc., Worcester, MA, USA) to calculate regional blood flow.

### Microcirculation

Regional blood flow was estimated using 15 μm neutron activated microspheres (BioPAL Inc., Worcester, MA, USA). The calculation [14] is based on the assumption that the microspheres are lodged in the capillaries [11] and that delivery of microspheres to a ROI depends on tissue blood flow. Tissue blood flow *u*_*tissue*_ (ml/min/g) is then calculated as:

$$ {u}_{tissue}=\frac{u_{ref}\cdotp {n}_{tissue}}{m_{tissue}\cdotp {n}_{ref}} $$

where u_ref_ is the reference sample flow, n_tissue_ represents the sphere count in the sample, m_tissue_ is the mass of the sample, and n_ref_ is the sphere count in the reference sample.

**Fig. 1:**
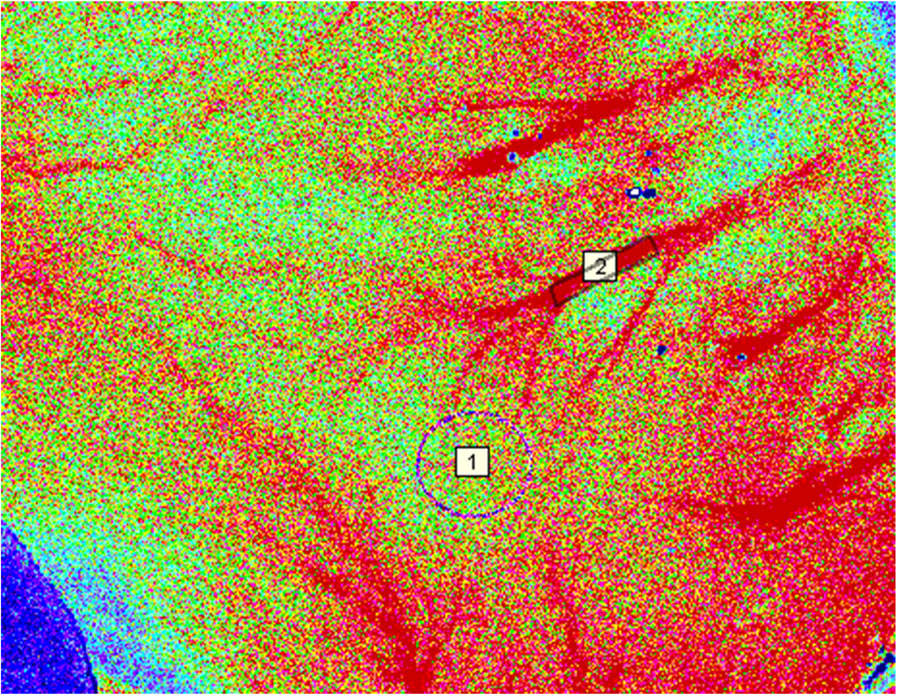
A representative illustration of the regions of interest as measured by laser speckle contrast imaging (at corpus of the stomach). 1: Corpus microcirculation; 2: An arteriole

The microcirculation was assessed with LSCI using a laser wavelength of 785 nm (MoorFLPI, Moor Instruments Ltd, Axminster, UK) placed parallel to the surface of the tissue and fixed 25 cm above each ROI. The ROIs were set to cover a 1 cm^2^ surface of the tissue to reduce variability of the measurements [15], with a sampling rate of 1 frame/s over 30 s. In contrast to microspheres, LSCI cannot measure absolute flow and results are expressed as flux in arbitrary laser speckle perfusion units which however are linearly related to absolute flow [12]. To further illustrate changes in blood flow following the induction of TEA, the flux at an arteriole adjacent to each ROI was determined post hoc using the software delivered by the manufacturer (Fig. 1).

### Statistics

Hemodynamic data represent the mean over 30 s recorded by the data acquisition software, whereas microcirculation at each ROI was extracted using a fixed algorithm in order to limit bias in selection of data [12]. Other statistic evaluations were calculated using IBM SPSS 20.0 (SPSS Inc., Chicago, IL, USA). Repeated measures were calculated using the nonparametric Friedman’s test with Bonferroni correction for multiple comparisons and Wilcoxon signed-rank test was used when comparing two time points. *P*-values <0.05 was considered statically significant and data are presented both as relative changes from baseline and absolute values as median with interquartile range (25th – 75th percentiles) for relevant interpretation of the findings.

## Results

**Fig. 2:**
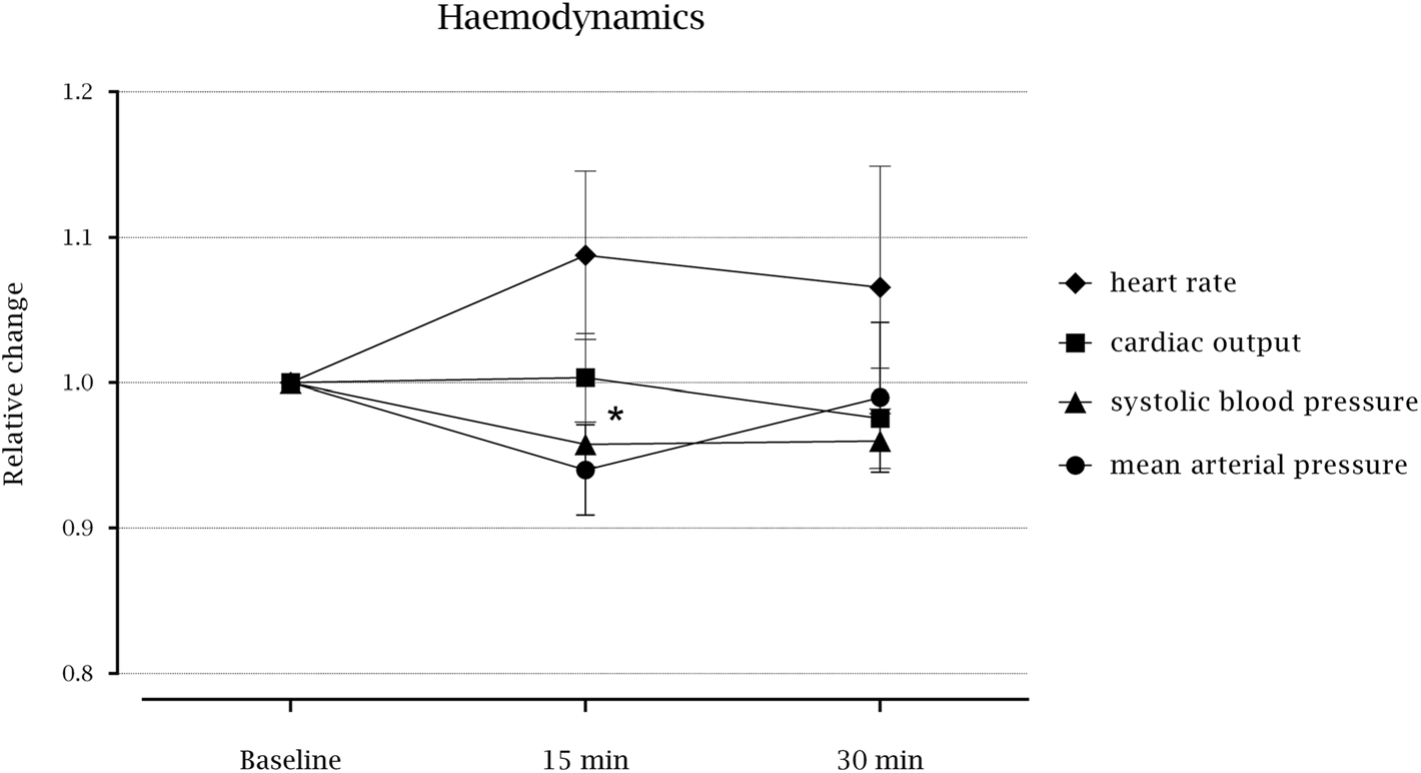
Median relative changes from baseline with interquartile range (25th – 75th percentiles) in haemodynamics following the induction of thoracic epidural analgesia (diastolic blood pressure not illustrated). * Decrease in systolic blood pressure 15 min following the induction (*p* = 0.03)

There was a decrease in systolic blood pressure following induction of TEA (*p* = 0.030), but no statically significantly changes in diastolic (*p* = 0.161) or mean arterial pressure (*p* = 0.154), cardiac output (*p* = 0.326), or heart rate (*p* = 0.236) (Fig. 2 and Table 1).

**Table 1:** Absolute hemodynamic values at baseline, 15 min, and 30 min after the induction of thoracic epidural analgesia, presented as median with interquartile range (25th – 75th percentiles)

Variable	Baseline	15 min	30 min
Systolic arterial pressure (mmHg)	115 (108–119)	104 (101–106)	105 (103–109)
Diastolic arterial pressure (mmHg)	66 (60–69)	59 (50–64)	60 (51–66)
Mean arterial pressure (mmHg)	85 (70–90)	74 (72–81)	76 (72–84)
Heart rate (beats min^−1^)	64 (57–76)	68 (58–89)	69 (53–84)
Cardiac output (ml min^−1^)	2800 (2550–3100)	2800 (2400–3350)	2600 (2400–3150)

### LSCI-determined arteriolar blood flow

**Fig. 3:**
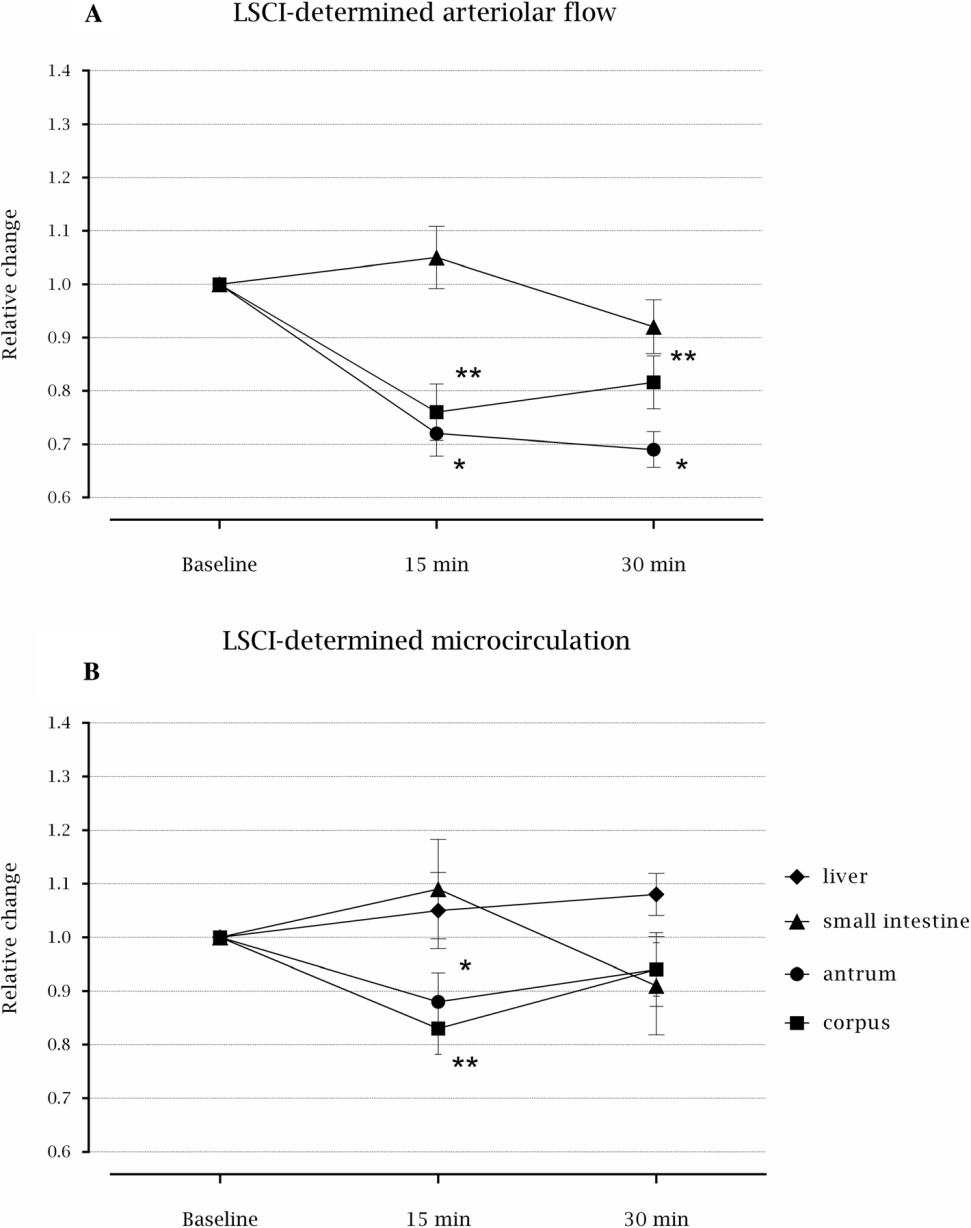
Median relative changes from baseline with interquartile range (25th – 75th percentiles) in microcirculation determined using LSCI following thoracic epidural analgesia at arterioles (**a**) and regions of interest (**b**). * and ** Decreased microcirculation at antrum and corpus, respectively (*p* < 0.05)

Fifteen minutes after the induction of TEA, flow at the arterioles decreased by 22 % at the antrum (*p* = 0.020) and by 19 % at the corpus (*p* = 0.029) of the stomach and the values remained low after 30 min (*p* = 0.001 and *p* = 0.012, respectively) (Fig. 3a and Table 2). In contrast, there was no significant change in arteriolar blood flow at the small intestine following the induction of TEA (*p* = 0.767).

**Table 2:** LSCI-determined arteriolar blood flow in laser speckle perfusion units at baseline, 15 min, and 30 min after the induction of thoracic epidural analgesia presented as median with interquartile range (25th – 75th percentiles)

Anatomic region	Baseline	15 min	30 min
Stomach: Antrum	1298.86 (1056.37–1464.11)	1019.52 (870.66–1168.86)	966.23 (811.12–1147.36)
Corpus	1164.43 (1000.18–1267.40)	944.39 (832.09–1194,16)	953.59 (773.97–1172.91)
Small intestines	1117.95 (1034.13–1243.63)	1252.54 (961.56–1346.93)	1220.18 (1008.93–1336.27)

### LSCI-determined microcirculation at regions of interest

In parallel with the changes in arteriolar blood flow there was a decrease in microcirculation by 19 % at the antrum (*p* = 0.015) and by 20 % at the corpus (*p* = 0.028) of the stomach following induction of TEA (Fig. 3b and Table 3) and these values were not significantly different from the decrease in arteriolar blood flow (*p* = 0.233 for antrum and *p* = 0.075 for corpus). The decrease in LSCI-determined microcirculation remained low, but was not statistically significant 30 min following induction of TEA (*p* = 0.062 for antrum and *p* = 0.091 for corpus). Also, there were no changes in LSCI-determined microcirculation after TEA for the liver (*p* = 0.115) or for the small intestine (*p* = 0.895).

**Table 3:** Absolute LSCI-determined microcirculation in laser speckle perfusion units at regions of interest, at baseline, 15 min, and 30 min after the induction of thoracic epidural analgesia, presented as median with interquartile range (25th – 75th percentiles)

Region of interest	Baseline	15 min	30 min
Stomach: Antrum	953.60 (700.90–1122.50)	777.30 (605.83–843.60)	796.60 (608.95–913.65)
Corpus	815.30 (679.20–910.77)	649.05 (622.25–711.38)	696.70 (622.80–90467)
Liver	442.30 (388.00–478.00)	414.65 (389.80–483.10)	458.00 (450.40–491.45)
Small intestines	937.40 (791.15–1033.75)	1019.95 (814.20–1121.35)	907.65 (727.60–1080.68)

### Regional blood flow assessed by microspheres

**Fig. 4:**
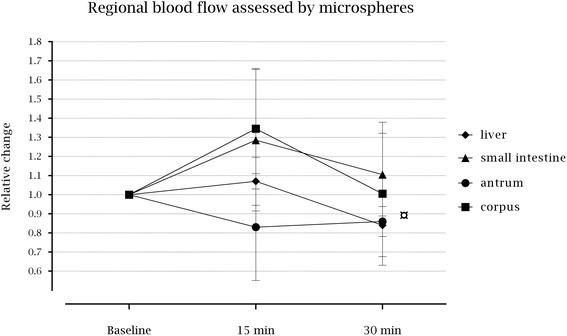
Median relative changes from baseline with interquartile range (25th – 75th percentiles) in regional blood flow assessed by microspheres following the induction of thoracic epidural analgesia. ¤ Decreased regional blood flow at antrum of the stomach 30 min following the induction (*p* = 0.048)

There was no decrease in regional blood flow to the antrum of the stomach 15 min after induction of TEA (*p* = 0.499) but after 30 min there was detected a decrease (*p* = 0.048). In contrast, regional blood flow to corpus of the stomach, liver, and small intestine did not change significantly (*p* = 0.417, *p* = 0.819, and *p* = 0.657, respectively) (Fig. 4 and Table 4).

**Table 4:** Absolute regional blood flow (ml/min/g) assessed by microspheres at baseline, 15 min, and 30 min after the induction of thoracic epidural analgesia, presented as median with interquartile range (25th – 75th percentiles)

Anatomic region	Baseline	15 min	30 min
Stomach: Antrum	0.20 (0.08–0.21)	0.14 (0.08–0.31)	0.11 (0.06–0.17)
Corpus	0.14 (0.05–0.23)	0.15 (0.08–0.24)	0.13 (0.09–0.28)
Liver	0.23 (0.10–0.33)	0.23 (0.75–0.52)	0.18 (0.05–0.40)
Small intestines	0.21 (0.13–0.34)	0.26 (0.17–0.59)	0.27 (0.17–0.40)

## Discussion

Without contact to the tissue, this study evaluated the effect of thoracic epidural analgesia (TEA) on liver, gastric, and small intestinal microcirculation by use of laser speckle contrast imaging (LSCI) and determination of regional blood flow by microspheres. In contrary to our hypothesis of maintained splanchnic microcirculation following the activation of TEA, microcirculation and blood flow in the arterioles appeared to decrease at both the antrum and corpus of the stomach following the induction of TEA and was accompanied by a drop in systolic blood pressure. Also, regional blood flow determined by microspheres at the antrum decreased following the induction of TEA, while flow to the liver and the small intestine was maintained.

Reduced systolic blood pressure and parallel decreases in microcirculation at the antrum and corpus of the stomach following induction of TEA are in line with studies in humans, indicating a TEA-mediated reduced splanchnic circulation [5, 6, 16–18]. Blockade of sympathetic nerve fibres with TEA results in peripheral and splanchnic vasodilatation and reduction in vascular resistance [19, 20], which we consider would increase the microcirculatory flow. However, the results indicate a reduced gastric microcirculation, while liver and small intestine microcirculation were sustained following induction of TEA. We cannot provide a simple explanation for these findings, but a rationalization may be the distribution and function of the intestinal vasculature with first- and second-order arterioles located in the serosal and submucosal layers, respectively, supplying a system of smaller third-order arterioles and terminal arterioles [21]. Each terminal arteriole supplies one or several villi, the muscle layer that overlays the perfused region and crypt regions associated with each villus [22]. The villi are sensitive to changes in oxygen supply and oxygen dependency may manifest with as little as a 30 % reduction in blood flow [23]. Therefore, it might be that blood is shunted away from the proximal to the distal part of the gastrointestinal tract (i.e., from the stomach to the small intestine) that has the largest number of villi. Such redistribution of flow could compensate for a reduced microcirculation to the villi by attenuated blood pressure and blood flow following induction of TEA. The stable microcirculation in the liver may be due to its complex blood supply with the largest part of flow derived from the portal circulation [24]. Splanchnic vasodilation following induction of TEA increases venous capacity, with subsequent increase in portal flow and therefore maintained liver microcirculation (the hepatic arterial buffer response) [25].

Reduced gastric microcirculation with TEA adds to the concern regarding the risk of anastomotic ischemia after, e.g., Ivor-Lewis esophagectomy where gastric continuity is re-established using the corpus of the stomach [26]. The integrity of an anastomosis depends on several factors, including surgical technique and viability of the muscle layers, but increased strain and local ischemia are believed to be main reasons for anastomotic leakage [27, 28]. A decrease in corpus microcirculation may question routine intraoperative use of epidural analgesia during esophagectomy, since diminished microcirculation has deleterious effect on the healing of the gastric conduit [4, 29]. An alternative may be that epidural anesthesia for postoperative analgesia after esophagectomy is activated only prior to the termination of general anesthesia.

The discrepancy between changes in regional blood flow as estimated by microspheres and microcirculation as assessed with LSCI likely reflects the different aspects of the microcirculation probed by the two techniques. The microspheres estimate a “snapshot” of absolute tissue blood flow rate (ml/min/g), while LSCI displays mean values of flux recorded (here over 30 s). Furthermore, the tissue samples obtained for calculations of regional blood flow were for the full wall thickness at the small intestine and stomach, as for samples from the liver. Thus, the microsphere-determined estimate of flow expresses blood flow rates through the whole sample thickness. LSCI on the other hand assesses the microcirculation to a depth of only approximately 1 mm [30]. This discrepancy suggests a compensatory reduction in microcirculation in serosal and muscular layers to counteract a reduced mucosal circulation following induction of TEA, as proposed by the increased mucosal microcirculation with TEA by Michelet et al. [9].

**Fig. 5:**
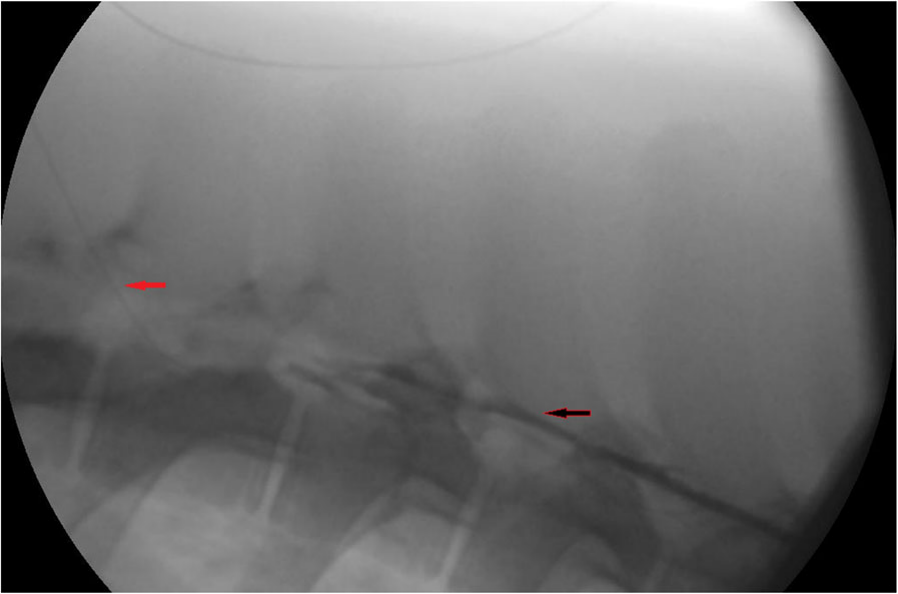
An epidurogram after the placement of the epidural catheter. Red arrow: The epidural catheter. Black arrow: Position documented using a contrast agent during fluoroscopy

Limitations of the present study include uncertainty regarding the precise position of the epidural catheters in pigs. Also, we did not demonstrate that the sympathetic nervous innervation to the splanchnic area was blocked by TEA since, e.g., plasma catecholamines were not measured. However, the “loss of resistance” technique for establishing epidural anesthesia is a clinical standard procedure with low rate of failure [31] and has been used previously in porcine studies [7]. Therefore, based on the changes in the hemodynamic values following the induction of TEA (i.e., drop in systolic blood pressure and (non-significant) increase in heart rate), we believe that the catheters were positioned correctly. Nonetheless, we accept that, e.g., an epidurogram [32] could be used to identify the position of the catheters, which was used in subsequent investigations (Fig. 5). Another limitation to the study may be the dose of the local anesthetics used for epidural blockage, since the volume used was small and the effect on pigs is not well known. The chosen volume was based on studies using TEA in pigs [7, 32] and deduced from the experience with the doses and concentrations used at our department. Further, the current results seem to indicate a delayed block to the lower part of the spine, presented as a (non-significant) decrease in microcirculation at the small intestine 30 min following induction of TEA. Therefore, the volume used may be insufficient and/or the evaluation was made too early to reveal the long-term effects of epidural anesthesia on the microcirculation. Also, the study setup with the present study conducted after another investigation [12] may have affected the microcirculation and its regulation. This may, therefore, not fully resemble the effect of epidural analgesia during clinical situations, with the epidural block often activated prior to surgery. Furthermore, tissue oxygenation of the splanchnic organs was not assed and tissue oxygenation could have been maintained despite a reduced blood flow. However, reliable techniques for assessing tissue oxygenation requires contact to [33] or penetration of tissue surface [7], which could affect the microcirculation. In addition, the objective of the current study was to assess the LSCI technique for monitoring splanchnic microcirculation during TEA, in which we believe, was achieved.

## Conclusion

The present study indicates that TEA may have an adverse effect on gastric microcirculation, as assessed by LSCI and microspheres in the pig. The results stress the need for randomized clinical trials to clarify the impact of TEA on gastric microcirculation. We suggest the non-touch technique offered by LSCI, to display changes in blood flow in real-time over a large area of tissue.
